# Effect of Pulsed Low-Level Lasers on Adult versus Neonatal Human Retinal Pigment Epithelial Cells: An *in-vitro* Study

**DOI:** 10.18502/jovr.v19i2.13278

**Published:** 2024-06-21

**Authors:** Seyed Mohamadmehdi Moshtaghion, Mohammad Abolhosseini, Faraj Tabeie, Sahar Balagholi, Fatemeh Suri, Samira Karami, Houra Naraghi, Mozhgan Rezaei Kanavi, Somayeh Asadi

**Affiliations:** ^1^Ocular Tissue Engineering Research Center, Research Institute for Ophthalmology and Vision Science, Shahid Beheshti University of Medical Sciences, Tehran, Iran; ^2^Department of Regeneration and Cell Therapy, Andalusian Molecular Biology and Regenerative Medicine Carre (CABINER) Seville, Spain; ^3^Department of Nuclear Medicine, School of Medicine, Shahid Beheshti University of Medical Sciences, Tehran, Iran; ^4^Blood Transfusion Research Center, High Institute for Research and Education in Transfusion Medicine, Tehran, Iran; ^5^Ophthalmic Research Center, Research Institute for Ophthalmology and Vision Science, Shahid Beheshti University of Medical Sciences, Tehran, Iran; ^6^Department of Hematology, School of Allied Medicine, Tehran University of Medical Sciences, Tehran, Iran; ^7^Department of Science and Engineering, University of Groningen, Netherlands; ^10^Seyed Mohamadmehdi Moshtaghion: https://orcid.org/0000-0002-6281-9674; ^11^Mohammad Abolhosseini: https://orcid.org/0000-0002-1914-4309; ^12^These two authors contributed equally to this work.

**Keywords:** Alpha-smooth Muscle Actin, Human Retinal Pigment Epithelial Cells, Low-level Laser, PAX6, Photobiomodulation, RPE65

## Abstract

**Purpose:**

To investigate the short-term effects of low-level lasers (LLLs; also known as low-power laser therapy) on the structure, genetic, and phenotype of cultured human retinal pigment epithelial (hRPE) cells from both adult and neonatal sources.

**Methods:**

Cultivated adult and neonatal hRPE cells were irradiated with two types of LLL (630 nm and 780 nm), 1 min daily for five consecutive days.

**Results:**

An increase in doubling time was observed in 630 nm-irradiated adult hRPE cells (*P* = 0.032). The gene expression profile revealed increased expression of retinoid isomerohydrolase *RPE65* (*RPE65*) (*P* < 0.01 for 630 nm laser, *P* < 0.001 for 780 nm laser) and nestin (NES) (*P* < 0.01 for 630 nm laser) in neonatal hRPE cells, upregulation of *RPE65* (*P* < 0.001 for 780 nm laser) and paired box 6 (PAX6) (*P* < 0.001 for 780 nm laser) genes in adult hRPE cells, and reduced expression of actin alpha 2 (*ACTA2*) in 780 nm-irradiated adult hRPE cells (*P* < 0.001). Except the significant increase of α -SMA in 780 nm-irradiated neonatal hRPE cells, no significant change was noted in the expressions of other investigated proteins.

**Conclusion:**

Short-term irradiation of neonatal and adult hRPE cells with LLLs may induce multipotency at the transcriptional level. Irradiation of neonatal hRPE cells with LLLs can be associated with increased risk of myofibroblastic transformation; however, adult hRPE cells irradiated with the 780 nm laser have minimal risk of myofibroblastic differentiation. It seems that the 780 nm laser may be a promising option for future photobiomodulation in retinal degenerations in adults.

##  INTRODUCTION

The single layer of human retinal pigment epithelial (hRPE) cells plays a pivotal role in upholding visual function by providing vital support to photoreceptor structural and functional integrity, as well as contributing to the maintenance of retinal homeostasis.^[1,2]^ Conditions such as age-related macular degeneration (AMD) and retinitis pigmentosa result from the loss or malfunction of hRPE cells, and as of now, effective treatments for these diseases remain elusive, leading to millions of cases of blindness worldwide.^[3]^ In pathological conditions, hRPE cells demonstrate a limited capacity for regeneration, emphasizing the imperative need to protect and ensure their continued maintenance and survival.

As an innovative treatment strategy, the restoration or substitution of deteriorated cells has been explored in various clinical trials implementing cultivated hRPE cells.^[4,5]^ In this regard, the implementation of the stem cells has been introduced as an effective therapeutic method for those patients with significant lack of photoreceptors. It has been shown that although neonatal stem cells have high potentials for retinal regeneration and replacement, they have ethical and regulatory considerations for stem cell therapies as well as carcinogenic potentials.^[6,7]^ It has been demonstrated that hRPE cells are capable of regeneration and trans-differentiation, so they can be considered as sources of retinal stem cell generation under some specific conditions.^[5,8]^ Although few studies have been done on these therapeutic advances for the diseases caused by hRPE dysfunction, there are still major concerns regarding the efficiency of the treatments and off-target differentiation of the implemented stem cells.^[9]^ Even in the most efficacious studies, only 0.1% of the transplanted cells could migrate to the desired locus.^[7]^

Optical-only stimulation has been shown to differentiate stem cells with stable lineage commitment that cannot further transdifferentiate.^[9]^ This indicates the potential of low-level laser (light) therapy (LLLT) in activating pathways in hRPE cells to eventually convert them to their stemness state and their further differentiation and substitution of the malfunctioned or destroyed hRPE cells. In this regard, Dang et al^[10]^ demonstrated that by using a continuous laser of 635 nm wavelength, the proliferative ability and expression of RPE-specific proteins of ARPE-19 cells were significantly increased; however, the trans differentiation and fibroblast-to-myofibroblast transition of the irradiated cells and the other types of LLLT were not addressed in their investigation.

LLLT is a fast-growing treatment technique which is widely used specifically in dermatology, cardiology, neurology, and orthopedics. It is a noninvasive light source treatment that generates a single wavelength of light in the visible to near-infrared range of 670–950 nm. This method provides pain relief and muscle relaxation as well as beneficial effects on wrinkles, acne scars, hypertrophic scars, and wound and burns' healing.^[11,12]^ The medical applications of LLLT have broadened beyond the superficial wound healings and chronic and acute pain relief, which include some diseases such as stroke, traumatic brain injuries, degenerative brain diseases, spinal cord injuries, and peripheral nerve disorders.^[13,14]^

LLL irradiation can induce alterations in gene products and cellular proteins via stimulating the mitochondria as the principle cellular photoreceptors.^[13]^ For instance, LLLT induces osteoblastic proliferation and differentiation in mesenchymal stromal cells, while it improves bony defects.^[15,16]^ In ophthalmology, this technique has been reported as an effective method for the treatment of retinal degenerative disorders such as retinitis pigmentosa and dry type AMD.^[17,18]^ It can also be used for protecting retinal photoreceptors against death in animal models.^[19]^

In this study, we compared the effects of short-term irradiation of adult and neonatal hRPE cells with 630 nm- versus 780 nm-pulsed laser in terms of cell proliferation, RPE-specific proteins, trans-differentiation, and myofibroblastic transformation.

**Figure 1:**
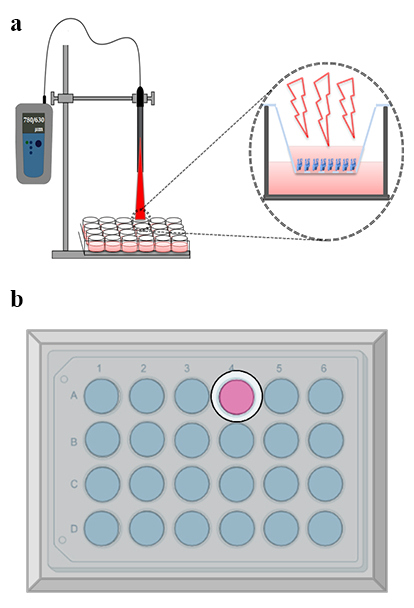
Laser irradiation experimental setup. (a) Illustration of the laser irradiation setup depicting the relative positions of the plate and laser diode. (b) Schematic representation of the plate covered with aluminum foil, featuring a 12 mm aperture designed to minimize scattering effects.

**Figure 2:**
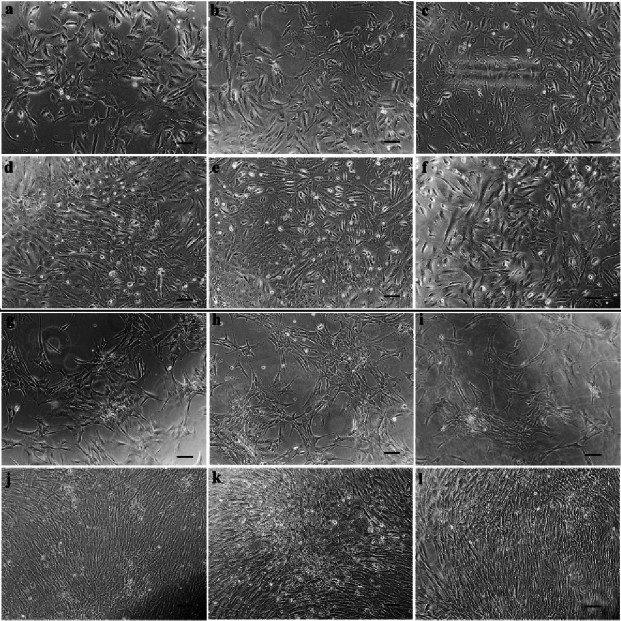
Adult and neonatal hRPE cells irradiated with 630 & 780 nm lasers. Note a mixture of spindle and polygonal morphology at baseline in adult cells irradiated with 630 (a) & 780 nm (b) lasers, and in neonatal cells irradiated with 630 (gr) & 780 nm (hr) lasers. (c) and (i) are the corresponding controls. After 5 days, adult cells irradiated with 630 (d) & 780 nm (e) lasers and their controls (f) still have a mixture morphology. Neonatal cells are predominantly spindle-shaped after a 5-day irradiation with 630 nm laser ( j), and polygonal after a 5-day irradiation with 780 nm laser (k) in comparison to controls (l) that show a mixture morphology. (Scale bar: 100 µm).

**Figure 3:**
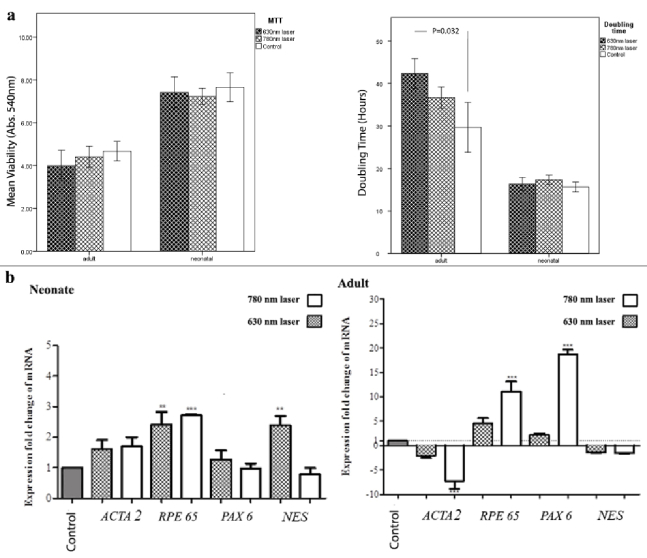
Viability, cell proliferation, and gene expression of adult and neonatal hRPE cells irradiated with 630 & 780 nm lasers. (a) MTT shows no significant difference between irradiated and control cells. 630 nm irradiated adult cells show a significant increase of doubling time (P=0.032) compared to control. (b) Note significant upregulation of RPE65 (P < 0.01 for 630 & P < 0.001 for 780 nm) and NES (P < 0.01 for 630 nm) genes in irradiated neonatal cells compared to control. In 780 nm irradiated adult cells, a significant increase of RPE65 and PAX6 (P < 0.001 for both) genes is observed compared to controls. ACTA2 gene in 780 nm irradiated adult cells is significantly downregulated (P < 0.001).

**Figure 4:**
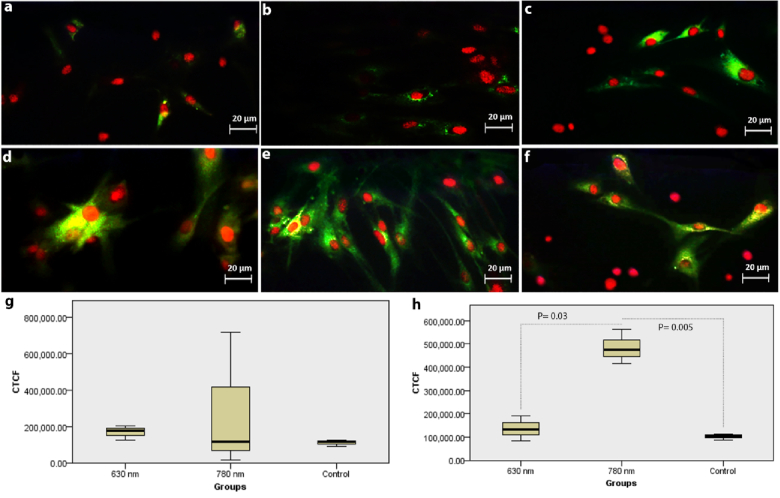
Immune reactivity for fluorescein isothiocyanate (FITC)-conjugated anti-α-smooth muscle actin (α-SMA) antibody in adult hRPE cells irradiated with 630 nm (a) & 780 nm laser (b), and in neonatal hRPE cells irradiated with 630 (d) & 780 nm laser (e) against controls cells (c & f). Propidium iodide-stained cell nuclei are red. Note no significant difference in expression of α-SMA between irradiated and non-irradiate adult cells (g) and significant increase of α-SMA in 780 nm laser-irradiated neonatal cells (P = 0.005) compared to controls, and compared with 630 nm irradiated neonatal cells (P = 0.03) (h).

**Figure 5:**
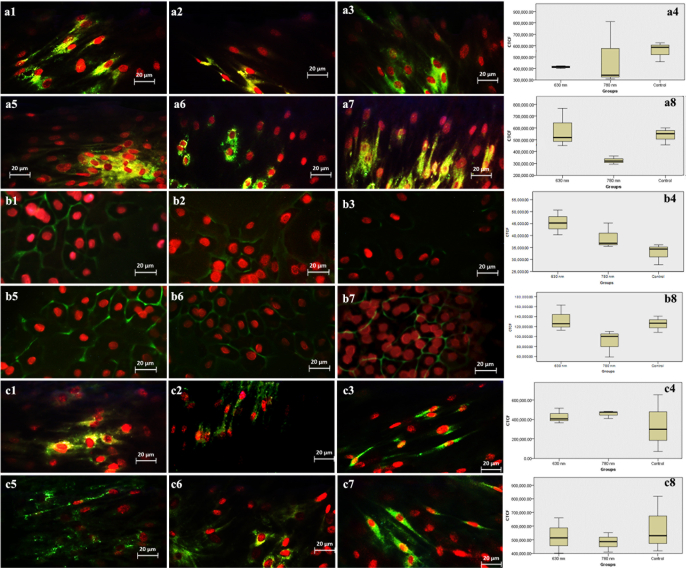
Immune reactivity for fluorescein isothiocyanate (FITC)-conjugated cytokeratin 8/18 (a), Na+-K+ ATPase (b), and nestin (c) in adult hRPE cells irradiated with 630 (a1,b1,c1) & 780 nm lasers (a2,b2,c2), and in neonatal hRPE cells irradiated with 630 (a5,b5,c5) & 780 nm lasers (a6,b6,c6) against controls (a3,b3,c3 for adult & a7,b7,c7 for neonatal cells). Propidium iodide-stained hRPE cell nuclei are in red. Note no significant alteration in the expression of the proteins between the study groups. (a4,b4,c4 for adult hRPE cells; a8,b8,c8 for neonatal hRPE cells).

**Figure 6:**
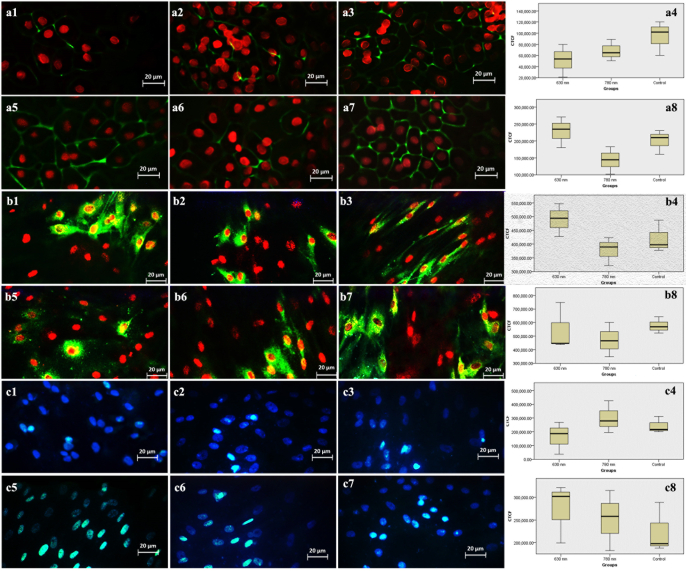
Immune reactivity for fluorescein isothiocyanate (FITC)-conjugated ZO-1 (a), RPE65 (b) and PAX-6 (c) in adult hRPE cells irradiated with 630 (a1,b1,c1) & 780 nm lasers (a2,b2,c2), and in neonatal hRPE cells irradiated with 630 (a5,b5,c5) & 780 nm lasers (a6,b6,c6) against controls (a3,b3,c3 for adult & a7,b7,c7 for neonatal cells). Propidium iodide-stained hRPE cell nuclei are in red except for PAX-6 in the nuclei are blue with 4’, 6 -diamidino-2-phenylindole (DAPI) (c series). Note no significant alteration in protein expressions between study groups (a4, b4, c4 for adult & a8, b8, c8 for neonatal cells).

**Figure 7:**
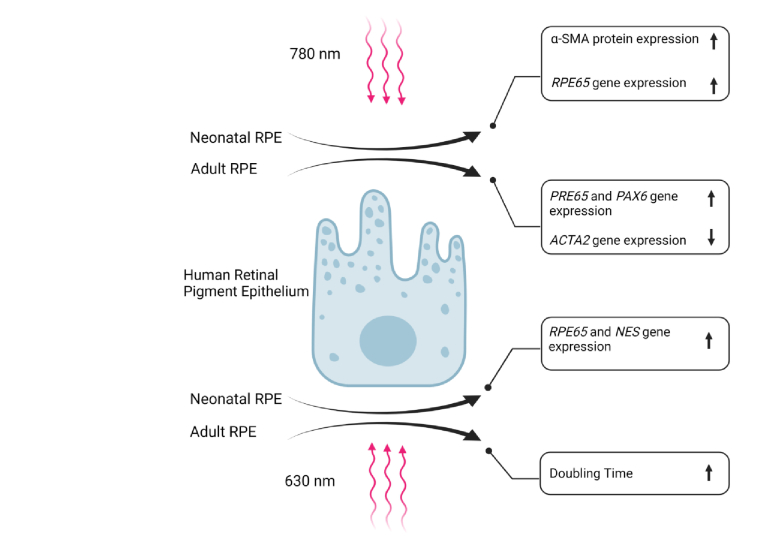
Graphical abstract illustrating the distinct effects of lasers at 630 nm and 780 nm on adult and neonatal hRPE cells.

##  METHODS 

The ethical approval was granted by the Ethics Committee at the Research Institute for Ophthalmology and Vision Science affiliated to Shahid Beheshti University of Medical Sciences, Tehran, Iran (IR.SBMU.ORC.REC.1397.7). The donated ocular tissues were used for performing this investigation in accordance with the Declaration of Helsinki regulations.

### Isolation and Cultivation of Adult and Neonatal hRPE Cells

Isolation and cultivation of hRPE cells were carried out using posterior eyecups obtained from two 40-year-old and two 1-month-old donors within 24 hr post-mortem, sourced from the Central Eye Bank of Iran. The acquired eyecups were divided into four quadrants, and the hRPE cells were isolated and cultivated following established protocols. ${\;}^{\left[20,21\right]}$ In brief, the dissected RPE layer was cut into small pieces and incubated in 2% dispase (Gibco, Godo Shusei Co., Ltd, Tokyo, Japan; 1 mg/ml) for 45 min at 37ºC. Subsequently, the digested tissue was centrifuged for 5 min at 300 gr at 4ºC and transferred into a T25 flask containing Dulbecco's modified Eagle's medium, Ham's F12 (DMEM/F12; GIBCO-BRL, Eggenstein, Germany), and 20% fetal bovine serum (FBS, GIBCO-BRL, Eggenstein, Germany). The cultivation took place in a humidified atmosphere with 5% CO ${\;}_{2}$ at 37ºC. The FBS percentage was gradually reduced from 20% to 10% as cell growth improved. Notably, experiments utilized cultivated hRPE cells at 90% confluency and passages 3–5.

### Application of LLLs on the Cultivated Adult and Neonatal hRPE Cells 

The LLLT system consisted of two diode lasers: one with the wavelength of 630 nm (visible) and another with the wavelength of 780 nm (near infrared). Both lasers had the power of 100 mW and the spot diameter of 12 mm. Lasers were operated at the pulse mode with the frequency of 35 Hz and the duty cycle of 50%. The power density per session for each laser was calculated to be 0.04 W/cm^2^ and by considering the irradiation time of 1 min, the corresponding energy density was 2.65 J/cm^2^.

The adult and neonatal hRPE cells were firstly passaged by trypsin/EDTA and then seeded on cell culture plates (Corning Costar, Corning, NY, USA) at the seeding density of 1 × 10^4^ per each DMEM: F12 culture medium. As well, low-level lasers (LLLs) with wavelengths of 630 nm and 780 nm were separately applied on the cultivated cells for 1 min per day for five consecutive days, with the cell culture plate lid removed during the irradiation of the laser to ensure the delivery of the maximum energy to the cells. The control groups consisting of the cultivated adult and neonatal hRPE cells received no irradiation. While performing the investigation, all cultivated plates were kept in darkness with aluminum foil coverage in order to minimize the effect of environmental conditions. At the time of the irradiation process, the laser was applied to the well of interest through a 12 mm-aperture that was made in the aluminum foil coverage. The control culture plates (no radiation groups) were allowed to stay in room light for 1 min per day during the study period. Figure 1 illustrates the schematic of the experimental setup for laser irradiation.

### Morphometric Evaluation of Cell Groups

Morphometric changes in all cell groups were evaluated and then photographed once at the baseline (before the irradiation) and once on day six (after the irradiation) using an inverted microscope (Olympus IX71; Tokyo, Japan) equipped with a digital camera (Olympus U-TV0.63XC; Tokyo, Japan).

### Survival and Proliferation of Cultivated hRPE Cells

At the end of the irradiation period (on day six), the viability and proliferation of the cultivated hRPE cells were determined using the 3-(4, 5- dimethylthiazol-2-yl)-2, 5-diphenyltetrazolium bromide (MTT) and doubling time (eq 1) tests, respectively. MTT assay was also performed as described earlier.^[22]^


$$mathrmdoublingtime=\frac{ln\;\left(2\right)}{gr}gr=\frac{ln\left(N\left(t\right)/N\left(0\right)\right)}{t},$$

where *N* (t) is the number of cells at time t, *N* (0) is the number of cells at time 0, gr is the growth rate and t is the time (hr).

Briefly, the hRPE cells in each group were seeded in 96-well plates at the density of 1500 cells/well in a final volume containing 100 μ l of DMEM: F12. After exchanging the cultured medium with 100 μ l of MTT solution (0.5 gr/ml, Sigma-Aldrich, Munich, Germany), the samples were incubated for 4 hours at 37ºC and 5% CO ${\;}_{2}$ until observing a purple precipitate. After the addition of 1 ml of dimethyl sulfoxide (DMSO) and incubating for 2 hours at room temperature in darkness, the obtained samples were read at 540 nm using an ELISA reader (EL × 808 Absorbance Reader, BioTek Instruments, Winooski, VT).

### Transcriptional Assay

Molecular investigations were conducted at the conclusion of the radiation exposure period, specifically on day six. Total RNA was extracted from both the irradiated and control (non-irradiated) groups. In brief, cellular lysis was achieved using TRIzol reagent (Life Technologies Corporation, Carlsbad, CA), followed by the addition of chloroform and isopropanol to precipitate the RNA. The resulting RNA precipitate was then dissolved in nuclease-free water. Subsequent to assessing RNA purity and concentration using a NanoDrop spectrophotometer (Thermo Fisher Scientific; Wilmington, DE) based on A260/280 values and concentrations, agarose gel electrophoresis was employed to confirm the integrity of both the 28S and 18S rRNA bands.

Utilizing a reverse transcriptase kit (Promega, USA) and oligo (dT) primers, the isolated RNAs were subjected to reverse transcription to generate cDNA. Furthermore, the expressions of the *ACTA2*, NES, *RPE65*, and PAX6 genes were quantified through quantitative real-time reverse transcriptase PCR using EvaGreen master mix (Solis BioDyne, Tartu, Estonia) and specific primers.

The PCR parameters included an initial amplification cycle for 10 min at 95ºC, followed by 40 cycles encompassing denaturation, amplification, and quantification (15 s at 95ºC, 30 s at 58–64ºC, and 25 s at 72ºC). A melting curve analysis, starting at 65ºC and gradually increasing to 95ºC was also performed. Expression levels were normalized to glyceraldehyde-3-phosphate dehydrogenase (GAPDH) as an internal control, and relative changes in gene expressions were analyzed using the 2- ΔΔ CT method, considering the standard curve and efficiency established for each primer set. All assessments were conducted in three independent experiments, with each sample run and examined in duplicate.

### Immunocytochemistry (ICC)

At the end of the radiation period (on day six), the hRPE cells in 24-well plates underwent fixation with cold methanol for 10 min, followed by permeabilization with 0.25% Triton X-100 (Sigma-Aldrich, Munich, Germany). Additionally, after blocking with 1% bovine serum albumin in PBS for 90 min at room temperature, the cells were subjected to incubation with the following antibodies overnight at 4ºC: anti-*RPE65* (1:200, rabbit polyclonal IgG; Santa Cruz Biotechnology, Dallas, TX), anti-cytokeratin 8/18 (1:200, mouse monoclonal IgG2a; Santa Cruz Biotechnology), anti-Na+/K+ ATPase (1:500, rabbit anti-human; Santa Cruz Biotechnology Inc., Dallas, USA), anti-Zo-1 (1:200, rabbit polyclonal IgG; Santa Cruz Biotechnology Inc., Dallas, USA), anti-nestin (1:400, rabbit monoclonal IgG, rabbit anti-human IgG; Santa Cruz Biotechnology), anti-PAX6 (1:500, goat polyclonal IgG; Santa Cruz Biotechnology), and anti-alpha smooth muscle actin ( α -SMA) (1:200 mouse polyclonal IgG; Santa Cruz Biotechnology Inc., Dallas, USA).

Following PBS irrigation, the secondary antibodies including fluorescein isothiocyanate (FITC)-conjugated goat anti-rabbit IgG (1:100; Santa Cruz Biotechnology Inc., Dallas, USA), FITC-conjugated goat anti-mouse IgG (1:100; Santa Cruz Biotechnology Inc., Dallas, USA), FITC-conjugated goat anti-rabbit IgG (1:200; Santa Cruz Biotechnology Inc., Dallas, USA), FITC-conjugated goat anti-rabbit IgG (1:200; Santa Cruz Biotechnology Inc., Dallas, USA), FITC-conjugated goat anti-rabbit IgG (1:100; Santa Cruz Biotechnology), FITC-conjugated donkey anti-goat IgG (1:100; Santa Cruz Biotechnology), and FITC-conjugated goat anti-mouse IgG (1:100; Santa Cruz Biotechnology Inc., Dallas, USA) were applied, respectively. This application was done on the cells that had been incubated with anti-*RPE65*, anti-cytokeratin 8/18, anti-Na+/K+ ATPase, anti-Zo-1, anti-nestin, anti-PAX6, and anti- α -SMA antibodies for 45 min at room temperature in darkness.

Except for the cells stained with anti-PAX6, which were counterstained with 4',6-diamidino-2-phenylindole (DAPI) (1 mg/ml; Santa Cruz Biotechnology Inc., Dallas, USA), other immune-stained cells were counterstained with propidium iodide (PI, 1 mg/mL, Molecular Probes Europe BV, Leiden, The Netherlands) for 5 min. Subsequently, all stained slides were examined using an inverted fluorescence microscope (IX81, Olympus, Tokyo, Japan) equipped with 535 and 617 nm filters for PI and FITC detection, respectively. Moreover, the corresponding images were captured using a digital camera (sCMOS, Neo 5.5sCMOS, ANDOR, Tokyo, Japan). Following this, ImageJ software (ImageJ 1.48; National Institute of Mental Health; http://rsb.info.nih.gov/ij/) was utilized to calculate the corrected total cell fluorescence (CTCF) of the immune-stained cells in each image.

### Statistical Analysis

To obtain the results of MTT and doubling time, Kruskal–Wallis and multiple comparison tests were both employed to evaluate the differences between the study groups. One-way analysis of variance (ANOVA) along with Dunnett's post comparison test (GraphPad; https://www.graphpad.com/) were performed to compare the results of RT-PCR and ICC between the study groups. *P*-value < 0.05 was considered as statistically significant.

##  RESULTS 

### Morphometric Alterations of Cell Groups

The cultivated adult and neonatal hRPE cells, either irradiated or non-irradiated, demonstrated a mixture of spindle-shaped and polygonal cells [Figure 2].

### Cell Viability and Proliferation 

MTT assay indicated no significant difference between the irradiated hRPE cells and the controls [Figure 3a]. However, an increase was observed in doubling time in the 630 nm laser irradiated adult hRPE cells compared to the controls (*P* = 0.032), showing the reduced cell proliferation rate in the corresponding irradiated cells. Although the doubling time in the 780 nm laser irradiated adult hRPE cells was higher than that of the controls, it was not statistically significant. Accordingly, doubling time assay demonstrated no significant change between the irradiated neonatal hRPE cells and the corresponding controls [Figure 3a].

### Gene Expression Profile 

The irradiated neonatal hRPE cells demonstrated the increased expressions of *RPE65 *(*P* < 0.01 for 630 nm laser, *P* < 0.001 for 780 nm laser) and *NES* (*P* < 0.01 for 630 nm laser) in comparison with the controls. A significant upregulation was also observed in *RPE65 *and *PAX6* (both *P* < 0.001) genes in 780 nm laser irradiated adult hRPE cells as compared to the controls. Notably, such genes' upregulation was not remarkable for the 630 nm irradiated adult hRPE cells. Although the expressions of *ACTA2* and *NES* genes reduced in the irradiated adult hRPE cells in comparison with the corresponding controls, it was only significant for the expression of *ACTA2 *gene in the 780 nm irradiated adult hRPE cells (*P* < 0.001) [Figure 3b].

### Immunocytochemistry

The results showed that except a significant increase that was observed in α -SMA in 780 nm laser irradiated neonatal hRPE cells (*P* = 0.005) compared to the controls and the 630 nm laser irradiated neonatal hRPE cells (*P* = 0.03) [Figure 4], no significant alterations were noted in the expression of the cytokeratin 8/18 [Figure 5a], Na+-K+ ATPase [Figure 5b], nestin [Figure 5c], ZO-1 [Figure 6a], *RPE65* [Figure 6b], and PAX-6 [Figure 6c] in the irradiated cells. Figure 7 represents a graphical abstract that illustrates the differential effects of lasers at 630 and 780 nm on irradiated adult and neonatal hRPE cells.

##  DISCUSSION

The findings of this study underscore the potential impact of short-term pulsed LLL irradiation on the proliferation and genotypic, and phenotypic traits of hRPE cells subjected to different wavelengths. In this context, our investigation revealed distinct responses to LLL irradiation between cultivated neonatal and adult hRPE cells. Specifically, the 630 nm laser treatment led to a reduction in the proliferation of adult hRPE cells, a phenomenon that could potentially be attributed to the pulsed nature of the LLL, as well as the substantial cumulative energy density delivered during the irradiation sessions.^[10]^ On the other hand, the 780 nm laser reduced the expression of *ACTA2* gene in adult hRPE cells; however, it induced no alteration in the expression of the corresponding α -SMA protein.

Of note, the transmission percentages for 630 nm red light and 780 nm near-infrared light across various ocular structures indicate that approximately 80–85% of the irradiated energy effectively reaches hRPE cells due to the wavelength-specific characteristics.^[23]^ Considering the reported effectiveness of LLLT in the treatment of retinal degenerative disorders, ${\;}^{\left[17,18,24,25\right]}$ its protective effects on degeneration of retinal photoreceptors,^[19]^ and based on the results of the current investigation, the application of the 780 nm laser can be regarded as a proper selection for adult subjects with retinal degenerative disorders.

Recent investigations in stem cell research have unveiled that human RPE cells undergo a multipotent stage during epithelial–mesenchymal transition. In laboratory studies, it has been observed that proliferative cells derived from human RPE express specific markers linked to the characteristics of stem cells. These findings hint at the potential of these cells, termed RPE stem cells, to possess stem cell-like properties.^[26]^ The expression of *PAX6* gene in the adult newts RPE cells and the expression of *NES* transcript affected by basic fibroblast growth factor in different vertebrate species and in human beings have shown that they can induce RPE reprogramming into the multipotency.^[27,28,29,30]^ In the current study, the increased expression of *PAX6* as well as the reduced expression of *ACTA2 *transcript in 780 nm laser irradiated adult hRPE cells indicated the high possibility of induced reprogramming and no risk of myofibroblastic transformation in the corresponding cells irradiated with 780 nm laser. The upregulation of *NES* transcript and unchanged expression of α -SMA protein under the irradiation with 630 nm laser in neonatal hRPE cells were the other possible evidence for reprogramming the neonatal hRPE cells into the state of multipotency. However, none of the corresponding groups disclosed any significant increase in the expression of PAX6 or nestin proteins or any decrease in the α -SMA protein in comparison to the controls. Correspondingly, this may be explained by the short interval of the study in which the proteins levels might be in a too low state to be detected in ICC studies. Therefore, further studies are needed to identify the proper irradiation duration for achieving the maximum effects on the expression of desired proteins.

In addition, the increased expression of some transcripts in the irradiated hRPE cells in the current investigation may possibly be explained by the ability of LLLs in altering gene expressions through modulating cellular metabolism as well as altering the transcription factors responsible for gene expression.^[31]^ A significant upregulation of *RPE65* gene in the irradiated neonatal hRPE cells (for both 630 nm- and 780 nm lasers) and in adult hRPE cells irradiated with the 780 nm laser, along with proper expressions of cytokeratin 8/18, *RPE65*, N ${\;}^{+}$ /K ${\;}^{+}$ ATPase, and ZO-1 proteins, all reflected the maintenance of characteristics and functionality of the irradiated hRPE cells.

Myofibroblastic transformation of hRPE cells is an undesired phenomenon, which clinically indicates the deteriorated nature of hRPE cells as well as the risk of fibrosis and proliferative retinopathy.^[30]^ Given that the 780 nm laser-irradiated adult hRPE cells expressed a significant downregulation of *ACTA2* transcript and no increase of α -SMA protein in comparison to those irradiated with the 630 nm laser, photobiomodulation with the 780 nm laser seems to be a more appropriate method in the adult subjects with RPE disorders, especially when this type of laser may induce reprogramming of the adult hRPE cells along with the increased expression of *PAX6* transcript. On the other hand, the results of 780 nm laser irradiation on neonatal hRPE cells showed a significant increase in α -SMA protein expression, which may make this type of laser non-suitable for the treatment of RPE disorders in infants. In such cases, the 630 nm laser may be considered as a more proper choice regarding the laser potential for the induction of reprogramming of the neonatal hRPE cells via the upregulation of *NES* transcript. The fact that whether longer durations of the LLLs irradiation attenuate or accentuate the aforementioned results needs further investigations.

In the current study, in contrast to the study by Dang et al,^[10]^ the proliferation of hRPE cells as well as the expression of RPE-specific proteins were increased by none of the implemented lasers. However, induction of trans-differentiation and multipotency at the transcriptional level were well demonstrated by both lasers in the neonatal and adult hRPE cells. This could be explained by the use of pulsed laser mode in our study versus the continuous mode used in the study by Dang et al,^[10]^ and also by the difference in the overall energy density. Low-energy densities like what were used in Dang et al's study^[10]^ might have had the potential for increasing the RPE cells proliferation but might not have been enough to stimulate cellular trans-differentiation.

One of the explanations for the efficacy of the LLLT is that the mechanism of action of the LLLT is indicated to be somehow similar to the optogenetics, which can be applied in both a low-dose light in form of laser or LED illumination to stimulate the corresponding chromophores. However, the chromophores utilized in LLLT are endogenous (mitochondrial) while the engineered light-activated chemical switches used in the optogenetics.^[32]^

In conclusion, our investigation, for the first time, showed that the pulsed LLLs with two different wavelengths of 630 nm and 780 nm could exert different effects on proliferation, and gene and protein expressions of the cultivated adult compared to the neonatal hRPE cells. However, both lasers were found to be capable of inducing trans-differentiation of hRPE cells to the retinal and neural progenitor cells at the transcriptional levels. The 630 nm laser reduced the proliferation rate of the adult hRPE cells, while the 780 nm laser significantly reduced the *ACTA2* gene expression of the adult hRPE cells and also preserved both the morphometric features and characteristics of the corresponding cells. Considering promising clinical results related to the application of photobiomodulation in various retinal degenerative conditions and based on the results of our investigation, the wavelength of 780 nm can be a proper option for application in further trials. However, further studies are needed to reveal the exact mechanism of action of LLLs and to identify the proper treatment duration for achieving the maximum photobiostimulation in the hRPE cells.

##  Financial Support and Sponsorship

None.

##  Conflicts of Interest

None.
